# Balloon-assisted coil embolization (BACE) of a wide-necked renal artery aneurysm using the intracranial scepter C compliant occlusion balloon catheter

**DOI:** 10.1186/s42155-018-0018-0

**Published:** 2018-10-03

**Authors:** Jeeban Paul Das, Hamed Asadi, Hong Kuan Kok, Emma Phelan, Alan O’Hare, Michael J. Lee

**Affiliations:** Department of Interventional Radiology, Beaumont Hospital and Royal College of Surgeons in Ireland, Dublin 9, Ireland

## Abstract

**Background:**

True renal artery aneurysms (TRAA) are an uncommon pathology, with a prevalence of less than 1%. Treatment of TRAAs is generally recommended when the aneurysm sac equals or exceeds 2cms. Both wide-necked and main renal artery branch aneurysms represent a challenge for conventional endovascular coil embolization due to the risk of coil migration.

**Main body:**

Intra-procedural remodeling of the aneurysm neck using Balloon Assisted Coil Embolization (BACE) is considered a suitable alternative in challenging cases of visceral artery aneurysms.

**Short Conclusion:**

We describe the novel use of the Scepter C (MicroVention Terumo, Tustin, CA) compliant double lumen neurovascular occlusion balloon in the treatment of a wide-necked branch TRAA in a patient with a solitary kidney.

## Background

True renal artery aneurysms (TRAA) are uncommon, accounting for 22% of all visceral artery aneurysms with a prevalence of less than 1% (Chung et al., 2016; Kok et al., 2016). Risk of rupture is low but increases significantly with enlarging aneurysm size, carrying a mortality rate of up to 80% (Kok et al., 2016). Treatment of TRAAs is therefore generally recommended when the aneurysm sac equals or exceeds 2cms (Chung et al., 2016). Wide-necked (> 4 mm) and main renal artery branch aneurysms represent a challenge for conventional endovascular coil embolization due to the risk of coil migration (Chung et al., 2016). Intra-procedural remodelling of the aneurysm neck using Balloon Assisted Coil Embolization (BACE), a technique adapted from interventional neuroradiology practice, is considered a suitable alternative in challenging cases of visceral artery aneurysms, and is growing in popularity compared to open surgery (Chung et al., 2016; Maingard et al., 2017).

We describe the novel use of the Scepter C (MicroVention Terumo, Tustin, CA) compliant double lumen neurovascular occlusion balloon to improve the safety and predictability of BACE treating a wide-necked branch TRAA in a patient with a solitary kidney.

## Main Text

**Fig. 1:**
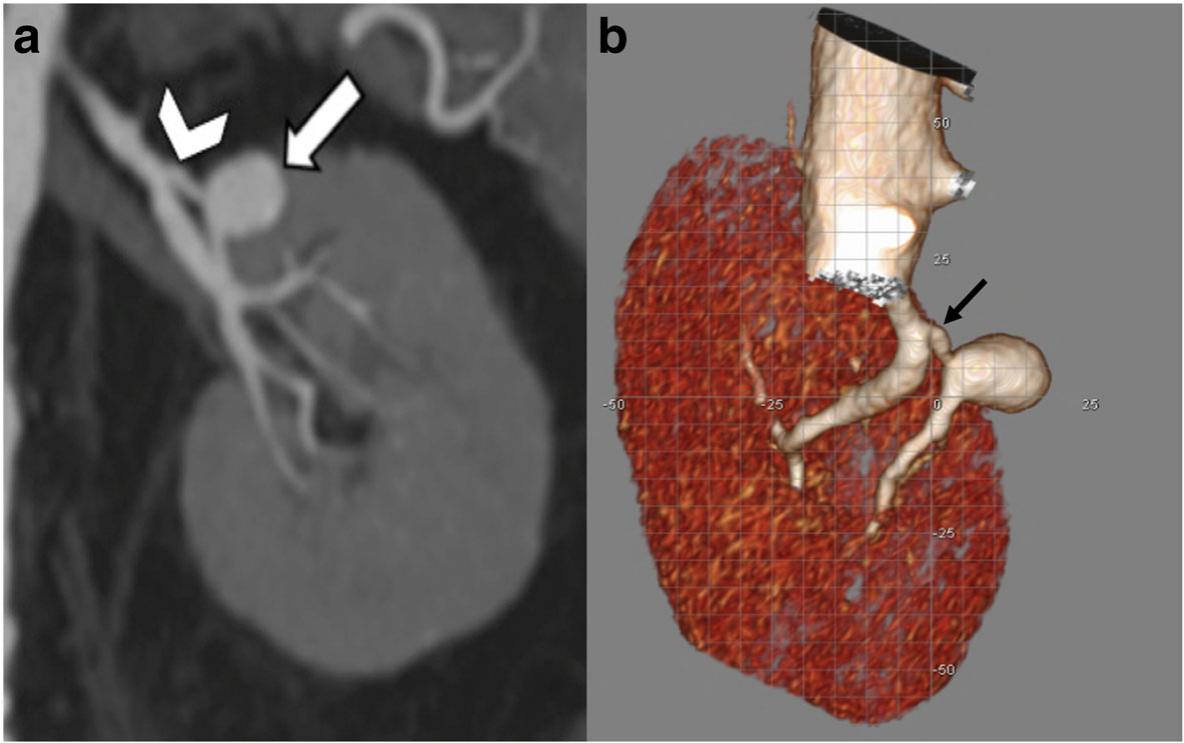
**a** CTA demonstrating a solitary left kidney with a wide-necked 2 cm saccular aneurysm (arrow) arising from the left renal artery anterior division (arrow head). **b** Volume-rendered 3D CTA reconstruction demonstrating the left TRAA morphology arising from the anterior division (arrow) just distal to the left renal artery bifurcation

A 64-year-old male who previously underwent right nephroureterectomy for transitional cell cancer presented for follow up of an asymptomatic TRAA. He had a medical history of hypercholesterolaemia, difficult to control hypertension and renal impairment with a serum creatinine of 120 umol/L (estimated glomerular filtration rate [eGFR] of 45 ml/min), a deterioration from his previously normal renal function measured 12 months previously. A CT angiogram demonstrated an interval increase in aneurysm sac size, now measuring 2 cm in maximum diameter (from 1.5 cm previously), with a relatively wide aneurysm neck of 8 mm (Fig. 1a). Maximum intensity projected reconstruction of the CT images revealed underlying fibromuscular dysplasia and better demonstrated the saccular aneurysm morphology, arising from the anterior division of the left renal artery, distal to the bifurcation **(**Fig. 1b**)**. In view of the increasing aneurysm size and refractory hypertension, a decision was made in consensus with the patient for endovascular treatment following discussion at a multidisciplinary meeting.

**Fig. 2:**
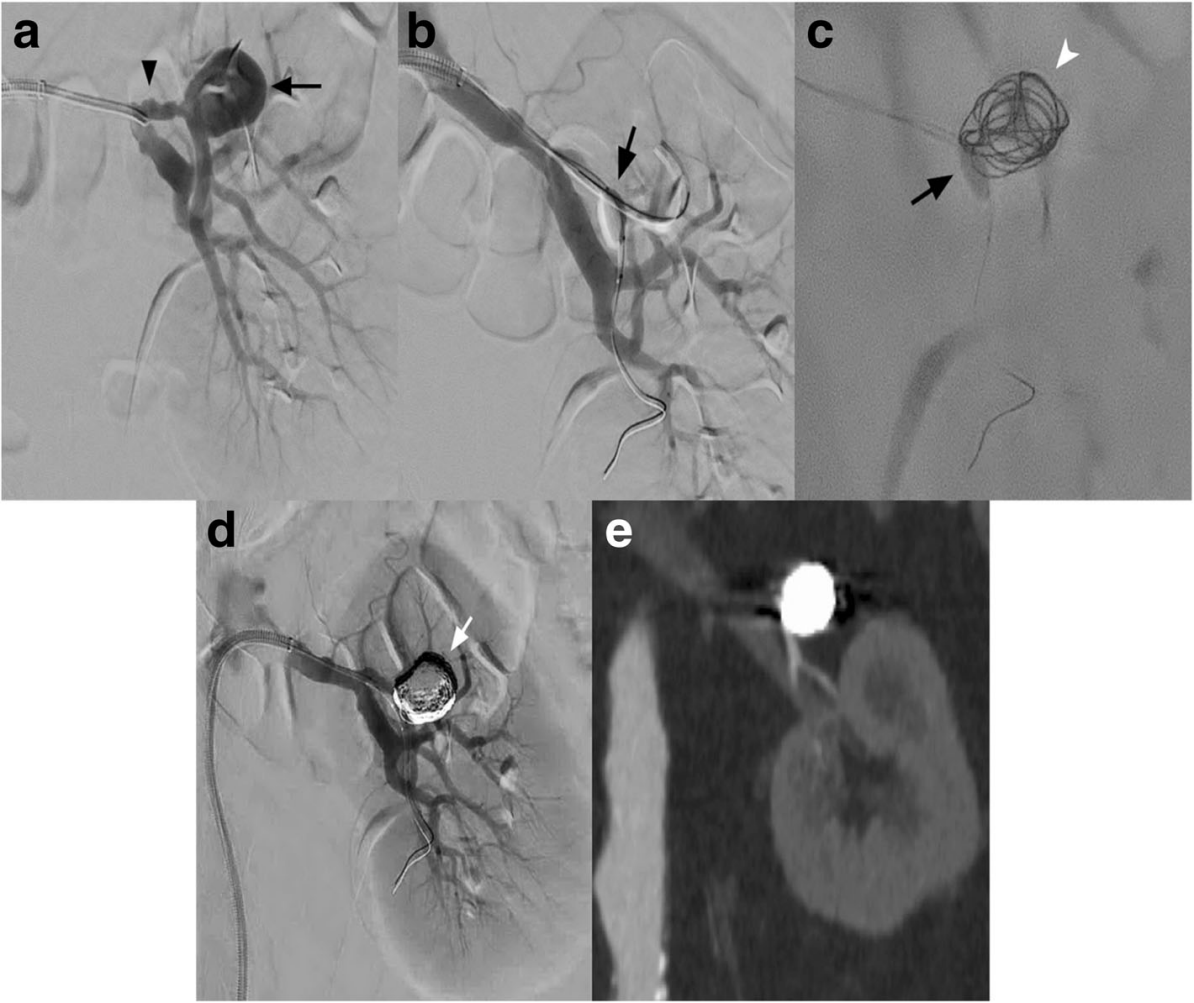
**a** Selective angiography of the left renal artery anterior division confirming the presence of a 2 cm wide-necked saccular aneurysm (arrow). The parent renal artery demonstrates a beaded appearance suggestive of fibromuscular dysplasia (arrow head). **b** Re-modelling of the wide-necked TRAA using the Scepter C balloon (arrow) which is inflated across the aneurysm neck. The coiling microcatheter tip has been positioned within the aneurysm sac. Angiography through the guide sheath shows occlusion of flow within the aneurysm sac and distal anterior division branch. **c** Fluoroscopic image demonstrating the inflated Scepter C balloon (arrow) with delivery of the initial detachable coil within aneurysm sac (arrow head). **d** Final completion angiography demonstrating complete obliteration of the aneurysm sac by a dense coil ball (arrow) with preservation of flow into the distal anterior division branch and renal parenchyma. **e** Follow-up CT angiogram at 3 months demonstrating persistent occlusion of the TRAA sac with normal solitary kidney parenchymal enhancement

Under conscious sedation and local anaesthesia, the right common femoral artery was accessed under ultrasound guidance and a 7-French guiding sheath (Destination; Terumo, Tokyo, Japan) was advanced into the abdominal aorta. The left renal artery was selectively catheterized using a 5F Cobra 2 catheter (Cook, Bloomington, IN) and the sheath was advanced into the proximal main renal artery. Angiography confirmed the CT findings of a 2 cm saccular aneurysm arising from the anterior division of the left renal artery with an 8 mm neck (Fig. 2a). A 2.8 French microcatheter (Progreat; Terumo) was advanced into the aneurysm sac and superselective angiography confirmed the absence of additional branches arising from within the sac. Concomitantly, a 4x10mm Scepter C occlusion balloon catheter (MicroVention) was prepared, de-bubbled and placed across the aneurysm neck. Following administration of 3000 units of heparin, the balloon was carefully inflated under fluoroscopic control to remodel the aneurysm neck and protect the renal vasculature (Fig. 2b).

Multiple detachable non-fibered coils (Axium Helical; Medtronic, Irvine, CA) were delivered to pack the aneurysm sac with the Scepter C occlusion balloon in-situ, preventing coil migration into the lumen of the left renal artery outflow vessel **(**Fig. 2c).

Final completion angiography demonstrated complete obliteration of the aneurysm sac, which was successfully excluded from the circulation, with preservation of blood flow to the distal arterial branch and renal parenchyma (Fig. 2d). There were no immediate peri-procedural complications and the patient was discharged home 48 h post-procedure. A follow-up CT angiogram 3 months later demonstrated persistent occlusion of the aneurysm sac with preservation of the parent vessel and renal parenchyma (Fig. 2e). His renal function normalised following the procedure, the most recent serum creatinine measuring 87 umol/L (eGFR 60 ml/min) and he remains well at follow-up.

## Discussion

Up to 75% of TRAAs are now treated via an endovascular approach. Benefits include lower morbidity associated with a less invasive procedure, better visceral preservation and lower incidence of complications, which can be as high as 18% in some surgical series (Kok et al., 2016). However, while trans-catheter coil embolization is a safe and effective treatment for narrow-necked saccular aneurysms, wide-necked aneurysms require a more sophisticated techniques such as balloon-assisted coil embolization as described above. This involves temporary inflation of a balloon across the neck of an aneurysm with an unfavourable neck anatomy to avoid the risk of coil herniation into the parent vessel and non-targeted coil embolization (Spiotta et al., 2013).

Embolization of wide-necked renal artery aneurysms in patients with a solitary kidney has been described using flow diversion techniques (Kok et al., 2016), covered stents (Kok et al., 2016) and neck-bridging devices (Maingard et al., 2017). BACE was selected as the preferred technique in this case to immediately occlude the aneurysm, while also preserving the arterial supply to the solitary kidney.

We elected to use a Transend EX 0.014-in. guidewire (Stryker Neurovascular, Fremont, CA) in conjunction with the Scepter C balloon to afford more steerability and more favourable navigation through challenging tortuous arterial anatomy (3,5). Increased steerability is further enhanced by the 5 mm length distal tip of the Scepter C making it easier for the balloon catheter to track across a sharply angulated vessel (Rho et al., 2013). Additionally, the use of a dual-lumen system for both neck remodelling and coil embolization may decrease the risk of thromboembolic complications due to the elimination of the need for two devices and the ability to use a smaller guide sheath or catheter for device delivery (Spiotta et al., 2013).

There are several limitations to BACE of aneurysms including, increased operative complexity owing to a greater number of guidewires and microcatheters required intra-procedurally and vessel dissection or rupture secondary to inflation of the balloon microcatheter in the vicinity of the aneurysm neck (Nelson & Levy, 2001).

The expense of detachable coils used for BACE procedures is also recognised as potentially prohibitive. Simon et al. (Relative cost comparison of embolic materials used for treatment of wide-necked intracranial aneurysms. Simon SD, Reig AS, James RF, Reddy P, Mericle RA, 2010) noted that for aneurysms between 3 and 25 mm in size, the financial cost of of embolization increased exponentially as the size of the aneurysm increased. For example, using Axium coils (eV3 Neurovascular) to embolize a 25 mm intracranial aneurysm could conceivably cost in excess of $50,000 to achieve an optimum packing density of 25%.

## Conclusions

In conclusion, endovascular treatment of TRAA is associated with excellent technical success, visceral preservation and a low rate of major complications (< 5%) and peri-procedural mortality of less than 2% (Kok et al., 2016). The use of neurovascular compliant balloons, like Scepter C, is a useful alternative to conventional single-lumen balloon occlusion microcatheters for BACE procedures and should be considered for challenging embolization cases where precise coil deployment is critical.
